# Review of nutrition management of pediatric intestinal pseudo‐obstruction

**DOI:** 10.1002/ncp.70115

**Published:** 2026-03-09

**Authors:** Senthilkumar Sankararaman, Raisa Rani James, Bushra El‐Amaireh, Andrea Adler, Kadakkal Radhakrishnan, Sujithra Velayuthan

**Affiliations:** ^1^ Pediatric Gastroenterology, Hepatology, and Nutrition Cleveland Clinic Children's Hospital Cleveland Cleveland Ohio USA; ^2^ Government Medical College Kozhikode India; ^3^ Jordon University of Science and Technology Irbid Jordan

**Keywords:** antroduodenal motility, CIPO, enteral nutrition, intestinal dysmotility, parenteral nutrition, PIPO, pseudo‐obstruction

## Abstract

Chronic intestinal pseudo‐obstruction (CIPO) is a rare, heterogeneous, and debilitating disorder characterized by profound intestinal dysmotility and severe nutrition challenges. Its presentation resembles that of mechanical bowel obstruction, but CIPO occurs in the absence of luminal obstruction. Pediatric‐onset CIPO has higher morbidity and mortality and is termed pediatric intestinal pseudo‐obstruction (PIPO) to differentiate it from adult‐onset CIPO. PIPO often presents with vague abdominal symptoms such as abdominal pain, distension, bloating, constipation, and diarrhea. Nutrition therapy is the mainstay of the management of PIPO. The main management goals include maintaining an adequate caloric intake, avoiding fluid and electrolyte imbalance, minimizing/managing malnutrition, treating gastrointestinal symptoms, enhancing intestinal motility using pharmacological interventions, and managing complications such as small intestinal bacterial overgrowth. Surgical interventions are reserved for severe cases. Various modalities of nutrition intervention include modification of oral diet, enteral feeding, and parenteral nutrition. Most children with PIPO have a poor quality of life, and the prognosis is variable based on the underlying condition. Prognosis is better in an interdisciplinary team setting in specialized centers of excellence.

## INTRODUCTION

Chronic intestinal pseudo‐obstruction (CIPO) is a rare gastrointestinal (GI) disorder characterized by intestinal dysmotility and severe nutrition challenges.^1^, ^2^ It is called intestinal pseudo‐obstruction as its presentation is very similar to true mechanical luminal obstruction but in the absence of obstructive pathologies.^3^, ^4^, ^5^ Even though its prevalence is low, CIPO is the second most common cause of intestinal failure after short bowel syndrome, accounting for approximately 15%–20% of all cases of intestinal failure.^6^, ^7^ The chronicity of CIPO is generally defined as the presentation of symptoms for ≥6 months (or >2 months from birth in congenital cases of CIPO).^4^

Based on the location and severity of GI involvement, the symptoms can vary between patients.^8^ The small bowel is almost always involved, followed by the colon and upper GI tract.^8^ Symptoms of CIPO can be vague, with most patients presenting with abdominal pain, distension, vomiting, diarrhea, and constipation.^5^, ^9^ Other presenting manifestations include bloating, nausea, gastroesophageal reflux disease (GERD) symptoms, altered bowel movements, feeding difficulty, and weight loss.^8^ As the above‐mentioned symptoms are not specific to CIPO and may overlap with more common etiologies (eg, disorders of gut‐brain interactions, lactose intolerance, celiac disease, and inflammatory bowel disease) a delay in diagnosis of CIPO is not uncommon.^8^ CIPO can also be mistaken for true intestinal obstruction (specifically the subacute form) given the clinical and radiological similarities.

Generally, pediatric‐onset CIPO is more challenging to manage and has higher morbidity and mortality when compared with adult‐onset CIPO. Additionally, there are key differences in CIPO between pediatric and adult populations with regard to etiology, clinical manifestations, diagnostic workup, treatment, and outcome. In 2018, the European Society for Pediatric Gastroenterology, Hepatology, and Nutrition (ESPGHAN)–led expert group recommended the term pediatric intestinal pseudo‐obstruction (PIPO) for pediatric‐onset CIPO to differentiate it from adult‐onset CIPO.^4^ Since then, PIPO is the preferred term and is often used to refer to CIPO in the pediatric population. Nutrition interventions remain the core aspect of PIPO, and various modalities include modification of oral diet, enteral feeding, and parenteral nutrition (PN). In this review, we detailed the overview of management of PIPO with a main focus on nutrition interventions.

## TREATMENT OF PIPO

**Figure 1:**
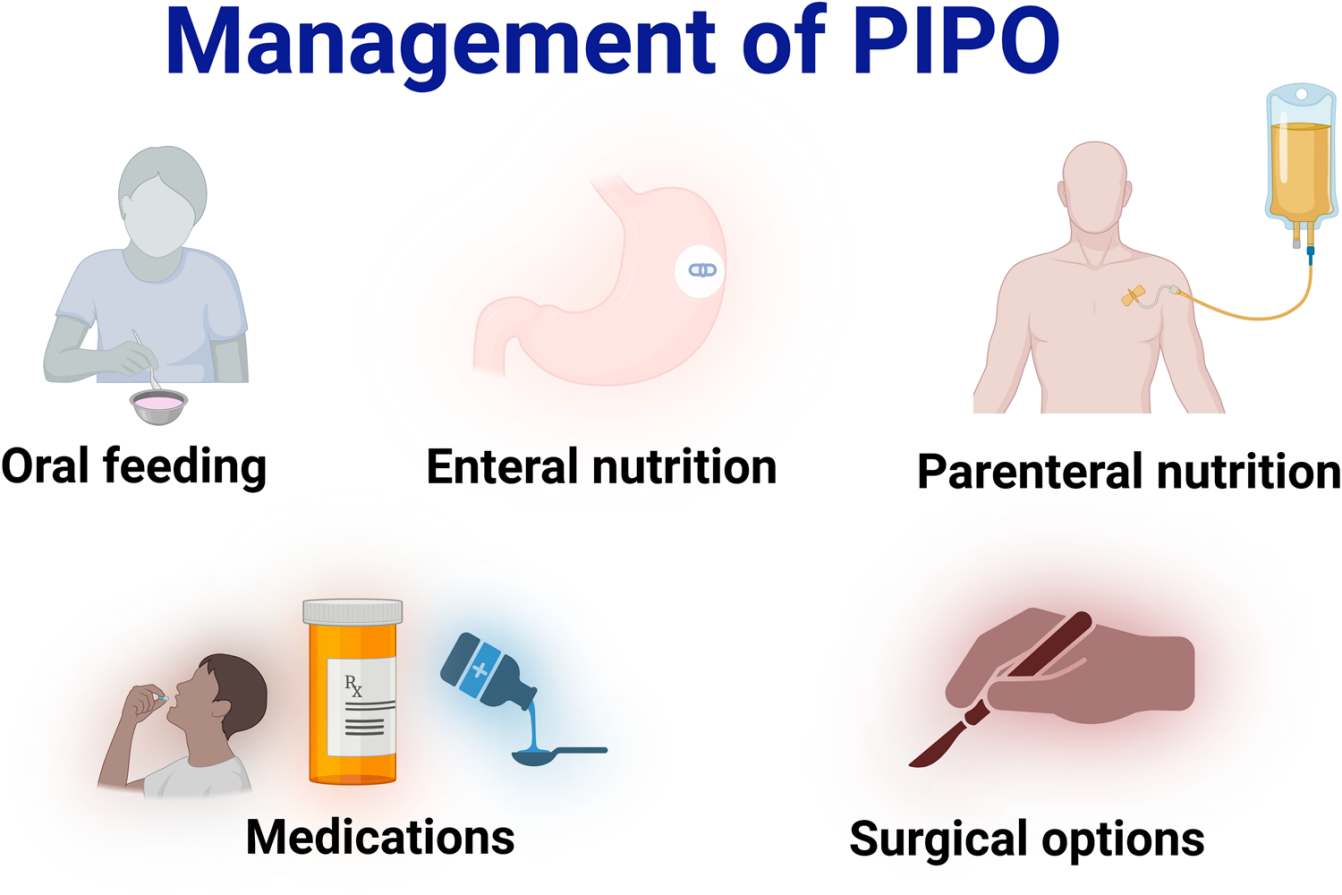
Overview of management options in PIPO. PIPO, pediatric intestinal pseudo‐obstruction. Created with BioRender.

The main management goal of pseudo‐obstruction include maintaining an adequate caloric intake, avoiding fluid and electrolyte imbalance, minimizing/managing malnutrition, treating GI symptoms, enhancing intestinal motility using pharmacological interventions, and also managing complications such as small intestinal bacterial overgrowth (SIBO)^10^, ^11^, ^12^ (Figure 1). Surgical interventions are reserved for severe cases.

### Nutrition management

Nutrition is the mainstay of the management, and careful and periodic nutrition assessment of each child with PIPO is mandatory. Patients with PIPO are generally malnourished for multiple reasons, such as decreased intake (owing to GI symptoms such as nausea, vomiting, and abdominal discomfort from dysmotility), impaired absorption, and increased losses (because of vomiting, diarrhea, or increased enterostomy losses). Patients who are severely malnourished are at risk of refeeding syndrome and should be carefully managed with slow initiation and advancement of feeds.^12^, ^13^, ^14^ A personalized and individual nutrition approach is needed to improve protein‐calorie malnutrition.^5^ Even in severe cases, unless contraindicated, maintenance of some oral diet for stimulation and retaining oral skills is important. Patients may develop an avoidant/restrictive food intake disorder resulting from the underlying condition along with intractable GI symptoms arising from dysmotility, such as early satiety, nausea, vomiting, and abdominal discomfort. Maintaining proper growth and development helps to promote motor function of the GI segments, leading to a better clinical outcome.^15^ Malnutrition in PIPO is illness‐related (secondary to inflammation/infection or dysmotility), non–illness‐related (caused by environmental/behavioral factors), or both.^16^ Further, impaired motility predisposes to SIBO, which in turn impairs nutrition absorption as well.^10^, ^15^

A careful assessment of the nutrition and caloric requirements of each child is important, and a therapeutic diet should be prescribed according to the needs. Treatment strategies should be precisely individualized based on the underlying etiology, comorbidies, enteral tolerance, and requirements. Nutrition can be administered via oral dietary modifications, enteral feeding, or PN (Table 1). An oral diet (however minimal it may be) is always the preferred initial intervention and might include bite and dissolve solids, liquid diet, or simple minimal oral stimulation in patients with severe symptoms.^17^ Dietary management should focus on diets that minimize symptoms and may also increase tolerance. If oral caloric intake is inadequate or not tolerated, enteral nutrition (EN), which can be nasogastric (NG) or gastrostomy (G) feeds, should be provided as the subsequent step. In the presence of severe gastric dysmotility, continuous postpyloric feeding (nasojejunal [NJ], gastrojejunal [GJ], or jejunal [J] feeds) are viable options. In the most severe cases, PN is required to meet appropriate nutrition requirements. Collating inference from many studies, approximately one‐third of patients with PIPO require oral feeding, one‐third require enteral feeds, and the remaining one‐third require PN.^4^, ^15^, ^18^ However, in patients with early‐onset PIPO diagnosed in the first year of life, two‐thirds of patients usually have a severe presentation and are dependent on PN.^29^

**Table 1:** Nutrition interventions in PIPO^4^, ^8^, ^15^, ^17^, ^18^, ^19^, ^20^, ^21^, ^22^, ^23^, ^24^, ^25^, ^26^, ^27^, ^28^.

Nutrition interventions	Different dietary options based on the severity of PIPO
General principles	– Evaluation and management of macronutrients and micronutrients – Optimization of hydration status – Gastrointestinal symptoms should be optimally controlled – Safety of oral feeding should be evaluated – Bone health should be optimized
Oral nutrition	– Small and frequent meals – Bite and dissolve solids – Small‐particle diet – Liquid diet – Low‐fat diet (<30% of total caloric intake) – Low‐fiber (low‐residue) diet – Low FODMAP diet – Formula/oral liquid supplements Polymeric formulas (during infancy, breast milk is strongly preferred over formula) – Supplementation of micronutrients
EN	– Gastric (bolus or continuous feeds) EN NG tube or G tube – Postpyloric EN ND tube or GJ tube and J tube – Formula types Polymeric Protein‐hydroxylate–based (casein‐predominant and whey‐predominant) Elemental formula Modular (carbohydrate and fat) additives
PN	– PN during hospitalization (transition from continuous to cyclic PN) – Home PN program – Evaluation and management of PN‐associated complications

Abbreviations: EN, enteral nutrition; FODMAP, fermentable oligo‐, di‐, mono‐saccharides, and polyol; G, gastrostomy; GJ, gastrojejunal; J, jejunal; ND, nasoduodenal; NG, nasogastric; PIPO, pediatric intestinal pseudo‐obstruction; PN, parenteral nutrition.

One multicenter, retrospective, observational study from the Netherlands on PIPO between 2000 and 2020 observed that 28 of 35 pediatric patients (80%) needed home PN (partial or total PN).^30^ In a study from China, 54 of 58 (93%) patients required PN during hospitalization, of whom 5 received PN alone and 49 received PN + EN.^12^ Home PN was not popular in this study, largely because of social concerns.^12^

In a survey from the European intestinal failure rehabilitation network, 73 children with PIPO from nine centers were included.^31^ Three‐fourths of the children became symptomatic in infancy. Nutrition practices in children with PIPO varied widely.^31^ Nine were on a normal diet, 64 (87.7%) required permanent nutrition support (2 patients received exclusive EN, 9 exclusive PN, and 53 received a combination of PN and oral diet [normal/bite and dissolve/normal but minimal intake] and/or EN).^31^ Nineteen patients (26%) eventually re‐established enteral/oral intake: 8 (42.1%) after stoma formation, 7 (36%) following prokinetic induction, and 1 (5.2%) after intestinal transplantation.

Beyond macronutrient and micronutrient needs, it is important to ensure that patients receive adequate hydration and electrolytes via the enteral (oral or EN) route or via PN.^17^, ^18^ Deficiencies of micronutrients and electrolytes, such as fat‐soluble vitamins (specifically D and K), vitamin B_12_, folate, calcium, iron, magnesium, and potassium, are widely prevalent in the CIPO/PIPO population.^2^, ^12^, ^15^, ^17^, ^19^, ^32^ These micronutrient deficiencies occur for many reasons, such as limited diet groups, restricted portion size, and impaired absorption.^11^ In a study by Tang et al, at baseline and during follow‐up, zinc deficiency rates were 29.6% and 26.3% and vitamin D deficiency were 26.9% and 52.6%, respectively.^12^ Calcium deficiency can be present because of the restriction of dietary intake of dairy and vegetables in this population, along with reduced absorption.^12^, ^19^ In patients with SIBO, vitamin B_12_ and folate deficiency can also be noted.^14^, ^19^ Tang et al also noted deficiency of vitamins B_1_, B_6_, C, and E either at baseline or during follow‐up.^12^ Chronic proton pump inhibitor (PPI) use in this population could worsen calcium, magnesium, and iron absorption. In patients who predominantly consume an oral diet, a multivitamin is recommended.^11^ Apart from a reduction in mortality and various morbidities associated with optimizing nutrition, improved nutrition in general also improves intestinal motility.^20^, ^21^, ^33^

#### Oral nutrition

The oral means of nutrition is physiological and is the preferred route. Whenever possible, oral nutrition should be encouraged and maximized based on tolerance. In addition to nutrition benefits, oral nutrition also provides opportunities for psychological benefits and an improved quality of life.^34^ Oral diet also provides social advantages of bonding among peers and family and increased satisfaction.^15^, ^35^ Safety of oral feeding should be investigated if symptoms or suspicion of oropharyngeal dysphagia exist.^15^ Infants and young children with feeding aversion and oropharyngeal dysphagia will be benefit from early referral to an interdisciplinary feeding team. Swallowing can be further objectively evaluated using a modified barium swallow test or using video fluoroscopy study. Children who have aspiration of feeds with thin consistencies will benefit from thickening the oral feeds.^17^ An ideal interdisciplinary feeding team should include a nutrition support physician, registered dietitian, speech‐language pathologist, occupational therapist, behavioral psychologist, nurse coordinator, and social worker.

Oral/enteral feeding is the most significant factor associated with improved prognosis in PIPO; hence, even patients receiving PN should be encouraged to take in as much oral/enteral feeding as possible.^20^, ^21^ The amount of oral intake in people with PIPO is determined by the extent of GI involvement.^11^ For children with pharyngeal and esophageal involvement, difficulties in suck and swallow may be encountered.^15^, ^36^ In children with delayed gastric emptying, symptoms such as abdominal pain, early satiety, bloating, and nausea with oral diet may be noted.^11^ Gastric emptying depends on multiple factors such as meal volume, osmolality, caloric content, and composition of the meal.^37^ Patients should be recommended to chew their food well.^14^ Liquid diet or formula/oral liquid supplement are alternatives if a solid diet is not tolerated well.^13^, ^38^, ^39^ Small and frequent (little and often) foods are preferred to improve tolerance, and liquid foods are tolerated better.^17^, ^19^, ^40^ Evidence‐based recommendations in PIPO are limited, and many of these recommendations are extrapolated from management of gastroparesis in adults because of similar and overlapping symptoms.^13^, ^15^ A registered dietitian experienced in managing people with PIPO is highly recommended.

#### Types of oral diet

Many dietary modifications such as bite and dissolve solids, small and frequent (six or more) meals, small‐particle diet, low‐fat diet, low‐fiber (low‐residue) diet, and low‐lactose or low–fermentable oligo‐, di‐, mono‐saccharides, and polyols (FODMAP) diet have been tried and tolerated based on the severity of PIPO.^11^, ^15^, ^38^, ^39^

##### Bite and dissolve solids

As the name denotes, these foods melt or soften readily, mixing with saliva and breaking apart easily with gumming, mashing, or minimal chewing.^41^ Some of the examples include wafers, cookies, crackers, puffed rice snacks, and cheese puffs.

##### Small particle‐size diet

Gastric antrum is primarily responsible for grinding, mixing, and trituration (process of reduction in size to <2 mm).^42^ Solid foods undergo trituration resulting in small particles before gastric emptying.^43^ Foods can be mashed with a fork or can be blended (pureed) using a blender to minimize the particle size.^13^ Some of the examples for easy‐to‐chew and swallow food include infant cereal products, blenderized vegetables, mashed potatoes or sweet potatoes, yogurt, mashed fruits (eg, banana or squeezable fruit products), and more options, which can be found in many reliable patient resource webpages.^44^, ^45^ These foods empty easily from the stomach and minimize the need for gastric accommodation.^41^, ^46^, ^47^

##### Low‐fat diet

A high‐fat diet delays gastric emptying; hence, a low‐fat diet is recommended.^15^ Medium‐chain triglycerides hasten gastric emptying and can be added with long‐chain triglycerides.^48^ Experts define a low‐fat diet as keeping calories <30% from fat.^8^

##### Low‐fiber diet/low‐residue diets

An oral diet may include solids, liquids, blended food, or chewable food depending on the extent and severity of gastric dysmotility. In case of gastric dysmotility, foods that tend to form bezoars, such as high‐residue (high‐fiber) and high‐fat foods (defined as >30% of total calories from fat), should be avoided. Foods with a high fiber content can cause delay in gastric emptying and bezoar formation, leading to increased transit time.^22^, ^23^ Low‐residue diets, which form less fecal matter, such as meat, chicken, fish, and white rice are preferred. Fibers are classified as soluble and insoluble fibers and both have an adverse effect on GI motility in patients with PIPO. Soluble fibers include alginate, pectin, and inulin, which delays gastric emptying.^13^, ^23^ High‐fiber foods include legumes/beans, whole grains, nuts/seeds, and most fruits and vegetables.^14^ A low‐fiber, low‐residue diet is helpful in intestinal gas (minimizing SIBO), cramps, and potential bezoar formation.^2^, ^11^, ^13^ Gas‐producing foods such as legumes and foods from the cruciferous family, such as broccoli, cabbage, cauliflower, kale, and Brussels sprouts, should be minimized.^24^

##### Low FODMAP diet

Foods rich in lactose, fructose, fructans, and other FODMAPs are minimized/avoided to help control symptoms such as bloating, cramps, distension, flatulence, and diarrhea.^17^ SIBO is another complication associated with the use of fermentable carbohydrates, especially in patients with dysmotility. Generally, avoiding the use of fermentable carbohydrates in irritable bowel syndrome (IBS) has been suggested as a method to avoid SIBO in IBS, but studies stating the same for PIPO are yet to be explored.

#### EN

Enteral feeds are given when oral feeding is inadequate or not tolerated to provide complete nutrition needs. In addition, fluid needs should also be evaluated and optimized with free water flushes. If the patient has minimal symptoms, different kinds of formulas (polymeric, protein‐hydroxylate, elemental, and blended) can also be used. However, in patients with severe symptoms, isotonic, low‐residue formulas with no fiber are generally used for feeding. Supplements with a higher proportion of medium‐chain triglycerides are preferred as they can accelerate gastric emptying and promote gut motility.^8^, ^25^ Caution should be used for home blenderized tube feeds to ensure appropriate consistency/smoothness and quality, and they should only be used under close medical supervision. In people with moderate to severe symptoms of PIPO, protein hydrolyzed formulas are often used and may be better tolerated than whole‐protein formulas because they are emptied from the stomach faster than casein or whole‐protein–based formulas.^11^, ^49^ Here, fiber‐containing formulas are generally not recommended to minimize GI symptoms.^13^ However, there is lack of high‐quality trials in this area.

##### Gastric feeding

Bolus feeding with small and frequent feeds is preferred as it is physiological. A trial of NG tube feeding is usually performed (but not always needed) to confirm GI tolerance before placing the permanent enteral access device, such as the G tube.^20^, ^21^ If the patient tolerates some oral feeds, then continuous feeds are given during the night hours, and the patient is allowed to feed orally (or bolus feeds if oral feeds are not feasible) during the daytime. However, when this is not tolerated, continuous feeds can be given for improved tolerance in patients with severe gastric dysmotility.^10^, ^26^ To enhance the calorie intake, a hypercaloric formula can be helpful if tolerated.^11^

##### Postpyloric feeding

In patients with gastroparesis or severe GERD, postpyloric (NJ, GJ, percutaneous endoscopic gastrojejunostomy, or J) tube feeds are better tolerated.^12^, ^26^ However, placement and maintenance of postpyloric tubes (NJ or GJ) are more challenging than gastric tubes (NG or G tube). A trial of NJ tube feeding is often preferred before recommending GJ but not mandatory.^38^ Even in patients receiving J tube feeding, a G tube is usually placed for venting to minimize PIPO symptoms.^27^ GJ tube has the advantage of a lower complication rate and also provides concurrent gastric venting.^38^, ^50^, ^51^ However, the jejunal limb can migrate retrograde back with retching and emesis into the stomach requiring reposition.^38^ In patients who have a GJ tube or separate G and J tubes, the G port can be used for venting.^14^ If patients do not tolerate J tube feeds, decompressive intestinal interventions such as colostomy or ileostomy can help in enteral tolerance before proceeding to PN. Di Lorenzo et al noted that detection of migrating motor complexes (MMCs) was associated with improved response to J tube feeding and a favorable prognostic marker.^26^ In that study, 18 children with PIPO who required PN or who did not grow while receiving G tube feedings were included. J tube feeding with an elemental formula was provided, and antroduodenal manometry (ADM) was performed between 2 months and 1 year after jejunostomy.^26^ Presence of MMCs in ADM was associated with better outcomes for J tube feeds.^26^

#### Electrolyte and fluid balance in high‐ostomy output

Electrolyte and fluid balance should also be managed to optimize the fluid and electrolyte losses in patients with surgical ostomies (specifically in jejunostomy or proximal ileostomy), which is done for decompression in a significant percentage of patients with PIPO.^52^ Acceptable ostomy output ranges from <30 ml/kg with an ileostomy up to <50 ml/kg with a jejunostomy.^53^, ^54^ The creation of an ostomy significantly impedes the absorption of chyme, leading to substantial loss of nutrients, water, and electrolytes.^55^ Because of a lack of specific literature in PIPO, the specifics of management can be inferred from literature detailing all populations who underwent intestinal ostomies.^55^ During episodes of acute dehydration and severe electrolyte imbalances, resuscitation and management should ideally be done in an inpatient setting (intensive care unit vs regular floor) to prevent further catastrophic outcomes such as acute kidney injury, cardiac arrhythmias, and neurological complications.^55^

For preventive strategies, dietary modifications should be aimed at minimizing ostomy output and improving stool consistency.^55^, ^56^ Low fiber intake is recommended to prevent intestinal blockage and high output.^57^ Ingested fluids should be preferably iso‐osmolar (~300 mmol/L) to plasma.^58^ Low‐osmotic (eg, water, zero‐calorie beverages, and tea) and high‐osmotic (eg, soft drinks) ingested fluids need to be restricted to avoid sodium and fluid losses, respectively.^58^, ^59^ In children with excessive output or poor weight gain, total body sodium can be evaluated using spot urinary sodium (aimed at >20 mmol/L).^58^ Small and frequent meals, starchy carbohydrates (eg, rice and pasta), and oral rehydration solution are encouraged in patients with PIPO to minimize the adverse consequences.^57^, ^60^, ^61^ A detailed approach of ostomy and high‐ostomy output has been discussed elsewhere.^52^, ^55^, ^56^, ^57^, ^58^, ^60^, ^62^, ^63^

#### PN

PN is initiated in patients with PIPO who fail oral and/or enteral feedings or in those who cannot meet their nutrition goals entirely by oral or enteral means.^9^, ^64^, ^65^ Many patients with acute episodes of PIPO may require PN during hospitalization or at home. However, many patients with PIPO (with or without oral and enteral feeding) may require home PN as the disease progresses.^30^ PN in this population is life‐saving, and a skilled interdisciplinary team is necessary to prescribe PN in the hospital as well as home‐based PN.^66^ As noted earlier, approximately one‐third of patients with PIPO will be dependent on long‐term PN, but prior studies have demonstrated a wide prevalence between 15% and 78%.^9^, ^29^, ^64^, ^65^, ^66^ The prevalence of long‐term PN depends on multiple factors, and increased prevalence is associated with the onset of PIPO (increased in neonatal and infantile presentation), history of megacystis‐microcolon‐intestinal hypoperistalsis syndrome, and prior pertinent surgical history.^9^, ^29^, ^64^, ^65^ Also, abnormal ADM findings (low‐amplitude phase 3 MMCs with a low motility index) were associated with PN dependence and higher mortality.^29^

In children, PN is initiated in the hospital setting as a 24‐h infusion, and slowly the duration can be gradually reduced to 12–20 h (cyclic PN) based on the age of the patient, risk of hypoglycemia, and presence of oral/enteral feeds. Cyclic PN infusion allows PN infusion‐free time and is more convenient for caregivers than continuous infusion.^67^, ^68^ Infants may tolerate only a few hours off PN, and older children and adults can ideally tolerate for 12 h as nocturnal infusion.^68^ However, abrupt initiation or discontinuation of cyclic PN, especially in children aged <3 years, may result in hyperglycemia or hypoglycemia, respectively.^67^ Additionally, gradual advancement of cyclic PN may improve or reduce cholestasis without increasing the risk of adverse metabolic effects.^67^, ^68^ Extensive parent/caregiver training for home PN is also provided before discharge. Individuals may experience serious complications from long‐term PN, including central line–associated bloodstream infections, venous thrombosis, and intestinal failure–associated liver disease (IFALD), and require management by an interdisciplinary advanced nutrition support team.^4^, ^20^, ^21^, ^69^, ^70^

Other PN‐related complications include imbalance (both excess and deficiency) in macronutrients and micronutrients, electrolyte abnormalities, abnormal glucose levels (hypoglycemia and hyperglycemia), poor bone health, and renal disorders (acute or chronic renal insufficiency and renal stones).^4^, ^12^, ^19^ Because of heterogeneity of conditions contributing to PIPO and a lack of specific high‐quality literature in nutrition management of PIPO, clinicians can carefully extrapolate relevant management strategies from pediatric intestinal failure caused by short bowel syndrome.^31^, ^58^, ^71^, ^72^ Implementation of an intestinal rehabilitation program has a significant impact on the reduction of septicemic episodes and survival of children with intestinal failure discharged on home PN.^73^, ^74^ Regardless of the underlying etiology, complications such as central venous line–related complications have already been extensively addressed in the American Society for Parenteral and Enteral Nutrition (ASPEN) and ESPGHAN resources.^75^, ^76^, ^77^ More research is needed in the PIPO population regarding the role of intravenous mixed lipid emulsions and different feeding approaches in the prevention of IFALD and antibiotic lock therapy to prevent other PN‐related complications. The management of PIPO is mainly supportive with nutrition interventions, as most prokinetic agents are unable to restore the normal motility of the GI tract.^11^, ^78^ Regardless, pharmacological interventions should be tried for all patients to evaluate the potential benefits.^11^

### Pharmacological interventions in PIPO

Medications include prokinetics and other medications for improving symptoms and complications.^5^ The commonly used prokinetic agents act either on the dopamine, motilin, and serotonin receptors, which are detailed in Table 2. Antispasmodics (eg, hyoscyamine and dicyclomine), antiemetic agents (eg, ondansetron, promethazine, antihistamines, and aprepitant), acid‐suppression medications (histamine‐2 antagonists and PPIs), and neuromodulators (tricyclic antidepressants and gabapentin) are commonly used to manage symptoms.^4^, ^18^ Rifaximin is not absorbed from the gut and is the preferred antibiotic for SIBO. Other antibiotics include amoxicillin/clavulanate, ciprofloxacin, doxycycline, metronidazole, tetracycline, and neomycin.^19^

**Table 2:** Medications used in PIPO^4^, ^10^, ^69^, ^79^, ^80^, ^81^, ^82^, ^83^, ^84^, ^85^, ^86^, ^87^, ^88^, ^89^, ^90^, ^91^, ^92^, ^93^, ^94^, ^95^, ^96^, ^97^, ^98^, ^99^, ^100^.

Medications	Mechanism of action	Side effects and cautions
Macrolides (erythromycin and azithromycin)^a^	– Act on motilin receptors in stomach and proximal intestine and increases gastric emptying and evoke MMC in the proximal small intestine – Compared with azithromycin, erythromycin is a stronger inhibitor of CYP3A4 enzyme and interacts more with multiple medications used concurrently – Tachyphylaxis is also common with macrolides	– Common side effects are nausea, vomiting, loose stools, and abdominal pain – Cardiac arrhythmias secondary to QTc prolongation has also reported when CYP3A4 enzyme inhibitors are used concurrently – Postnatal exposure to erythromycin has been associated with increased incidence of hypertrophic pyloric stenosis in infants, and the association is stronger if the exposure occurs in the first 2 weeks of life
Amoxicillin‐clavulanate^a^	– Accelerate small bowel transit by evoking high amplitude and prolonging phase 3 of the MMC	– Common side effects include nausea, vomiting, loose stools, and abdominal pain
Somatostatin analogue (octreotide)	– Increases phase 3 of the MMC in intestine and inhibits gastric activity. Increases frequency of MMCs in small intestine by binding to type 2 receptors – Improvement in symptoms have been noted in both children and adults with CIPO – Can be administered as an intravenous infusion or as subcutaneous injections	– Side effects include allergic reaction, hyperglycemia, cholecystitis, gallstone pancreatitis, and hypertension. Decreases gastric emptying
Metoclopramide	– Antagonizes central and peripheral D2 receptors in the medullary chemoreceptor trigger zone in the area postrema. Also, it an antagonist of 5HT3 and an agonist at 5HT4 receptors and accelerates gastric emptying. It also increases the resting tone of the lower esophageal sphincter while at the same time relaxing the duodenal bulb and pyloric sphincter and therefore augments the peristalsis of the duodenum and jejunum	– Metoclopramide is sparingly used because of the black box warning – Extrapyramidal side effects such as tardive dyskinesia (irreversible), cardiac arrhythmias (in patients with long QT interval on EKG), diarrhea, and sedation
Domperidone	– Domperidone does not cross the blood‐brain barrier and acts on the peripheral D2 receptors – It also acts on the chemoreceptor trigger zone, which is not protected by the blood‐brain barrier and exerts its antiemetic effect	– With domperidone, QTc prolongation has also been reported. In the US, it is only available for use in patients aged >12 years who have failed standard therapies through an expanded access investigational new drug program. It is, however, widely used in other countries
Prucalopride	– Highly selective 5HT4 receptor agonist that lacks the cardiac side effects of its predecessor, cisapride. 5HT4 receptors are expressed largely in the epithelium of the colon and less extensively in the small bowel. Activation of these receptors by prucalopride increases serotonin and fluid secretion and increases intestinal peristalsis – Also causes increased lower esophageal sphincter resting tone, enhanced esophageal clearance, gastric emptying and colonic transit. It is effective in both gastroparesis and CIPO	– Headache, nausea, abdominal cramps, and diarrhea are the common side effects. The most serious side effect of this medication is sudden cardiac death because of prolongation of the QTc interval – The risk of QTc prolongation increases when it is used concomitantly with other medications that causes QTc prolongation, such as erythromycin and ondansetron
Velusetrag^b^	– Velusetrag is a potent, newer medication with a very selective 5HT4 agonist that is being investigated. A phase 2 trial in patients with chronic constipation and diabetic and idiopathic gastroparesis has shown promising results in improving CIPO symptoms	– The common adverse events noted in these studies are diarrhea, headache, nausea, and vomiting
Neostigmine and pyridostigmine	– Neostigmine and pyridostigmine prevent degradation of acetylcholine in the synaptic cleft by the acetylcholinesterase enzyme. They increase availability of acetylcholine in the synaptic junction. Action on M2 and M3 results in decreased transit time in the gut. Subcutaneous administration of neostigmine improves colonic motility in patients with Ogilvie syndrome. Pyridostigmine as a longer acting and oral form is available as well	– Nausea, vomiting, abdominal cramps, diarrhea, sialorrhea, muscle cramps, miosis, urinary frequency, bronchial hypersecretion, and bradycardia are common side effects. Bronchoconstriction is a known side effect of cholinomimetic drugs and should be used in caution in patients with asthma and chronic pulmonary disease. Bradycardia is another common side effect, and patients should be monitored with cardiac monitor when neostigmine is being administered
Bethanechol	– Bethanechol is a cholinergic agent that increases esophageal and antral motility by acting as an agonist at the muscarinic receptors. In people with ineffective esophageal motility, administration of oral bethanechol increased distal esophageal contraction and improved bolus transit	– Nausea, vomiting, abdominal cramps, diarrhea, sialorrhea, muscle cramps, miosis, urinary frequency, bronchial hypersecretion, and bradycardia are expected cholinergic side effects

Abbreviations: 5HT, 5 hydroxytryptamine; CIPO, chronic intestinal pseudo‐obstruction; CYP, cytochrome p450; D, dopamine receptor; EKG, electrocardiogram; M, muscarinic receptor; MMC, migrating motor complex; QTc, corrected QT.

^a^ Medications used by general pediatric gastroenterologists. Other medications are commonly used by neurogastroenterology and motility specialists.

^b^ Please note this is not US FDA approved.

### Surgical management

Surgical management in PIPO is done for both therapeutic and palliative purposes, and the requirement of surgical intervention depends on the severity. In the Dutch PIPO study, approximately 90% had bowel surgeries, including formation and/or closure of venting ostomies.^30^ Surgical interventions include rectal biopsy (partial‐thickness biopsy in younger infants and full‐thickness biopsy in older children), feeding and decompressing enterostomies, gastric electrical stimulation, and intestinal and multivisceral transplantation.^30^, ^49^ If dietary manipulations and medications fail to improve the symptoms, venting gastrostomies, feeding jejunostomies, and/or cecostomies can be used.^19^ A retrospective study of CIPO in adult patients showed that patients underwent surgery for localized dysmotility with resection and bypass and ostomies (gastrostomy and enterostomy) for feeding and decompression.^101^ This study reported decreased hospital admission and improved quality of life after patients underwent surgical management.^101^ In myopathic patients with PIPO, even minimally invasive surgery has the risks of postsurgical sequelae and prolonged recovery of bowel function after surgery.^78^

Some experts recommended subtotal colectomy with ileorectal anastomosis for a small number of patients with PIPO who experienced frequent enterostomy‐related complications.^102^ Severe liver failure, loss of venous access, or catheter‐related sepsis were noted as indications of multivisceral transplant.^27^, ^103^, ^104^ Studies have shown that patients with malrotation, short bowel syndrome, urinary tract involvement, and myopathies demonstrated worse outcomes.^105^, ^106^ Also, surgical interventions increase the risk of paralytic ileus in the postoperative period and also increase the risk of secondary mechanical intestinal obstruction.^30^ Stoma prolapse has also been frequently reported in PIPO.^107^ Hence, surgical planning should be carefully done after discussion with the interdisciplinary team input, and centralization and standardization of care for this complex rare disease is recommended.^12^, ^30^, ^49^, ^66^ Rarely, stoma reversal might be considered in a minority of people with PIPO who have long‐standing stable disease but minimal symptoms (eg, ≥2 years without pseudo‐obstruction episodes, no EN/PN requirement, and minimal bowel dilatation).^78^

### Prognosis

Most children with PIPO have a poor quality of life, and the prognosis is variable based on the underlying predisposing condition.^108^ Patients with PIPO generally have a poor quality of life because of frequent admissions, clinic visits, emergency department visits, and surgical interventions.^30^ Management options and prognosis are variable among the patients based on the underlying etiology.^30^ Central venous catheters and feeding tubes also create difficulties in performing normal everyday tasks like other healthy children and are associated with a restricted quality of life.^108^ Long‐term PN is associated with complications, such as central line–related problems (central line occlusion and catheter‐related bloodstream‐related infections) and other metabolic complications such as PN‐associated liver disease.^12^, ^30^ Absence of oral feeding, long‐term PN, prolonged hospitalization, and other factors were noted with increased risk of catheter‐related bloodstream‐related infections in infants with PN.^109^ Hence, initiation and advancement of enteral feeding as soon as possible should be attempted. In prior studies, the prognosis with successful intestinal rehabilitation and achieving enteral autonomy was generally lower in PIPO when compared with short bowel syndrome.^110^, ^111^ Poor outcomes are reported in infants and young children with PIPO; patients with very short remaining bowel, presence of stoma, and myopathic PIPO; and patients with urologic involvement.^9^, ^12^, ^33^, ^65^, ^66^ Myopathy was suggested as a predictor of mortality in children with primary PIPO.^65^ Many advancements in targeted gene sequencing approaches in the diagnosis of PIPO are being developed, and in the near future clinicians can expect targeted revolutionary therapies such as gene editing in the management of PIPO.^112^

## CONCLUSION

CIPO is a severe GI motility disorder with a multitude of etiologies and is characterized by recurrent episodes of abdominal distension and other symptoms of intestinal obstruction with no mechanical obstruction. The main focus of CIPO management is to optimize nutrition; preserve, and when possible, improve GI function and motility; minimize GI symptoms; and treat the underlying conditions and complications. Various modalities of nutrition therapy include modification of the oral diet, enteral feeding, and PN. Along with nutrition interventions, other core principles of management include medications to augment intestinal motility and surgical interventions for severe cases. An interdisciplinary team involving, for example, a gastroenterologist, surgeon, dietitian, pharmacist, registered nurse, social worker, and psychologist is essential for the proper management of patients with PIPO.

## AUTHOR CONTRIBUTIONS

Senthilkumar Sankararaman contributed to the conceptualization, original draft, and review and editing. Raisa Rani James contributed to the conceptualization, original draft, and review and editing. Bushra El‐Amaireh contributed to the conceptualization, original draft and review and editing. Andrea Adler contributed to the supervision and review and editing. Kadakkal Radhakrishnan contributed to the supervision and review and editing. Sujithra Velayuthan contributed to the supervision and review and editing. All authors approved the final draft.

## CONFLICT OF INTEREST STATEMENT

Senthilkumar Sankararaman is a consultant for Nestlé. The remaining authors declare no conflict of interests.
